# Delineation of partial melts and crustal heterogeneities within the crust beneath Kumaon Himalaya, India from L_g_ wave attenuation

**DOI:** 10.1038/s41598-023-36269-z

**Published:** 2023-06-19

**Authors:** Mahesh Prasad Parija, Sudesh Kumar, Arjun V H

**Affiliations:** 1 CSIR-National Geophysical Research Institute, Hyderabad, India; 2 Department of Geosciences, Virginia Tech, Blacksburg, VA USA; 3 National Center for Polar and ocean Research, Ministry of Earth Sciences, Headland Sada, Vascodagama, Goa 403804 India

**Keywords:** Seismology, Natural hazards

## Abstract

The crustal seismic attenuation or the Q structure is studied by using the Fourier spectra of Lg-wave along the Tanakpur- Dharchula- Dharma transect in the Kumaon Himalaya. The 1 Hz Lg Q (Q_0_) values are computed between different pairs of two stations and the observed values are later utilized to calculate the lateral variation in the Q_0_ values by following a back projection algorithm. This computation of Q_0_ values utilizes five regional distance earthquakes having moment magnitude (Mw) ≥ 4.0, which lie along the great circle path of the transect. Three of the five earthquakes occurred in the Tibetan plateau and the and the others occurred to the southwest on the Indian shield and are well recorded at all the 32 broadband seismographs operated between September 2018 and March 2022. The estimate Qo values range from 63 ± 2 and 203 ± 25, with the lowest value in the Lesser Himalaya and the highest across part of the Indo Gangetic Plain and Siwalik Himalaya. The Q_0_ model has low values ∼200 along the profile in the Indo Gangetic Plain and the Siwalik Himalaya, and are correlated with 2–5 km thick sedimentary layers below the Himalaya and the adjoining Indo-Gangetic Plain. We observe two distinctly different Q_0_ values to the northeast in the Lesser Himalaya tectonic unit. The region lying between the South Almora Thrust (SAT) and the Berinag Thrust (BT) shows extremely low Q_0_ values (∼60) but increases further north towards the Vaikrita Thrust (VT) to ∼200. The possible explanation for observing such huge variation of the Q_0_ values within a single tectonic unit may be the presence of fluid rich ramp structures, which introduces crustal heterogeneities and traps the aqueous fluids or partial melts lying within the crust. The Lg Q_0_ values decrease to the North and become ∼166 for station pairs in the Higher Himalaya and Tethys Himalaya tectonic units. The low Q_0_ values observed in this region may be correlated with low viscous partial melts in the form of Miocene leucogranite plutons, which resulted out of the Indo-Asian collision. The attenuation structure along the profile in the Kumaon Himalaya can be used to estimate ground motions of future earthquakes in the area and can contribute to seismic hazard assessment in the Himalaya and neighbouring regions.

## Introduction

The Lg wave is a super critically reflected S-wave, travels within the continental crust, between the free surface and the Moho^1,2^ and is the most prominent seismic phase recorded at regional to teleseismic distances provides an important insight on nature of lithology and its tectonic environments^3^. The attenuation of Lg wave vary significantly across major continents due to the presence of partial melting, fluid contents and different compositions of rocks from one region to another^4^. In several continental regions where there are considerable fluctuations in crustal thickness, such as the mountain belts in central Asia, the Tibetan, the Bolivian Altiplano and close to the Alpine ranges, strong attenuation of Lg has been reported. In tectonically active areas such as Tibet, scattering of Lg energy from fractures within the shallow crust, partial melting and low S-wave velocity layer within the crust may be a significant factor for strong Lg attenuation^5–9^. The values of Lg Q vary up to an order of magnitude from tectonically stable (> 700) to unstable regions (< 200)^3,10^. There have been significant studies worldwide to characterize the attenuation property of a medium through Quality factor (Q) estimation utilizing different types of seismic waves for e.g. coda-wave, Lg-wave, P-wave and S-wave, etc. recorded at local and regional distances from an earthquake location^4,11–19^. The effect of fluid or melt fraction is more dominant in attenuation property (1/Q) compared to the S-wave velocity. While the S-wave velocity in crust varies by less than 20%, Q varies by a factor of 3 on major continents^20^.

The present profile, which lies in the Kumaon sector of the northwest Himalaya stretches to about 200 km in length along the northeast-southwest direction. The transect cuts perpendicular across the major fault structures in the region that have a strike directing northwest-southeast.

**Figure 1:**
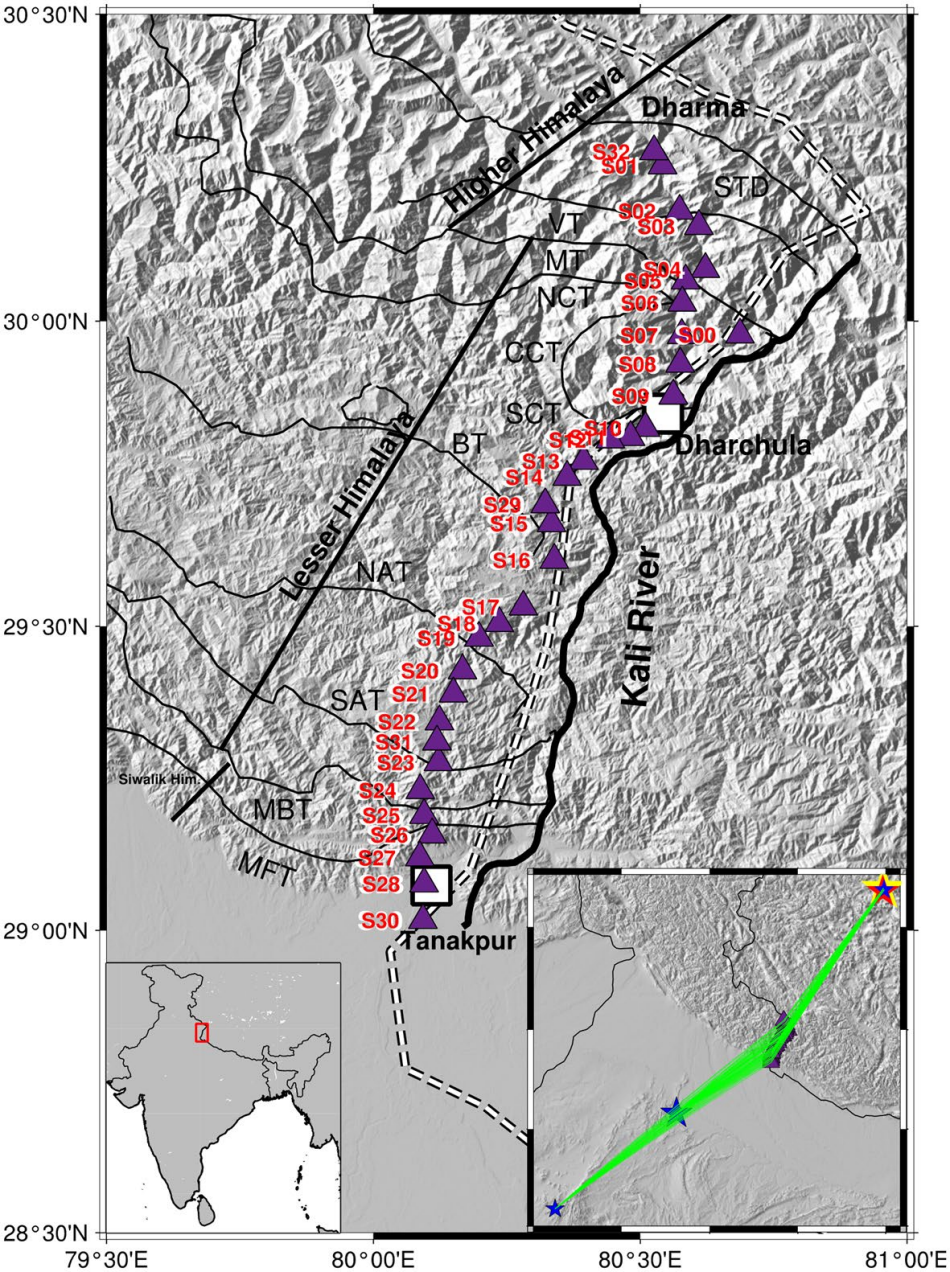
Regional tectonic map of the Kumaon Himalaya showing major thrust systems. *MFT* Main frontal thrust, *MBT* Main Boundary Thrust, *SAT* South Almora Thrust, *NAT* North Almora Thrust, *BT* Berinag Thrust, *SCT* South Chippalkot Thrust, *CCT* Central Chippalkot Thrust, *NCT* North Chippalkot Thrust, *MT* Munsiari Thrust, *VT* Vaikrita Thrust, *STD* Southern Tibetan detachment. Broadband seismographs operated during 2018–2022 is marked as filled triangle (blue). Inset 1 shows the Indian map with the study area marked as a red square. Inset II shows the geographical location of the five regional earthquakes (solid star) used in analysis and the recording stations (filled triangle). Three earthquakes in the Tibet are represented in the form of solid stars with different sizes and colours. Rest two earthquakes from Indian shield are represented as solid blue star. The figure has been generated using the Generic Mapping Tool (GMT), version 6.0 (https://docs.generic-mapping-tools.org/6.0/).

The present seismic profile consists of stations that are fit by the great-circle path under a least square criterion^3^ and thus provide us with the opportunity to invert for the lateral variation of Lg Q along this best-fit profile in the Kumaon section of the northwest Himalaya (Fig. 1). We have utilized the interstation *Q*_0_ computation approach^3^ to image the attenuation across the major structural features of the Kumaon Himalaya that extends from the Indo-Gangetic plain in the south across the Main Frontal Thrust (MFT) to the north of the South Tibetan Detachment (STD) fault in the Tethys Himalaya. The first step involves calculation of the interstation Q_0_ values from the selected pair of stations. The interstation Q_0_ values are then used to map the lateral variations of *Lg Q*_0_ using a back projection method. We find low Lg Q_0_ along the entire length of the profile, which we interpret in terms of crustal ramps and /or trapped partial melts, which supports the outward channel flow of the Tibetan crust. Attenuation measurement is important in investigating the presence of aqueous fluid/partial melt in the crust, and can provide constraints on the quantification of ground motions from future earthquakes in the Himalaya and neighbouring regions.

## Seismotectonics and previous geophysical studies

The spectacular Himalaya, mountain chain resulted from the northwards underthrusting of the Indian plate with the Eurasia since 50 Ma^21,22^. The subduction of the Indian plate beneath the Himalaya caused lateral deformations giving rise to fault structures from north to south. These crustal scale faults have a NW–SE strike and are designated as the Indus Tsangpo Suture Zone (ITSZ), the Main Central thrust (MCT), the Main boundary thrust (MBT) and the Main frontal thrust (MFT)^23,24^. The MFT bounds the Himalaya from the adjoining Indo-Gangetic Plain (IGP) to its south. Four different tectonic units referred to as the Siwalik Himalaya; the Lesser Himalaya; the Higher Himalaya and the Tethys Himalaya are separated from each other by thrust faults at the respective southern and northern boundaries by the MFT, MBT, MCT, STD and ITSZ respectively (Fig. 1). The other major thrust faults between the MBT and the MCT are the Almora thrust which is further divided into a north Almora thrust (NAT) and south Amotha thrust (SAT) and the Barinag Thrust (BT). These surface faults penetrate in depth to about 5 km^25^.

The western Kumaon region lies in the central seismic gap (CSG) and several geological and geophysical studies suggest a ramp structure involving the Main Himlayan Thrust (MHT) at mid-crustal depth that is responsible for major and great earthquakes in the Himalaya^26^. Recently, crustal investigations using receiver function analysis along the Kali River of the Kumaon Himalaya have shown variations in the crustal thickness of ~ 38 km to 41 km from the Indo-Gangetic Plain (IGP) to the Higher Himalaya and have also detected partial melts at mid-crustal depths^27^. A study to the west of the Kali river region^28^ found a flat-ramp-flat structure of the MHT and a low shear wave velocity zone (LVZ) beneath the Lesser Himalaya between the Main Frontal Thrust (MFT) and the Main Central Thrust (MCT). This LVZ is well corroborated by low resistivity found in magnetotelluric studies^29,30^. Receiver function analysis on the eastern side of the Kali river in Nepal reported that the Moho beneath western Nepal is gently dipping northward, from a depth of ~ 40 km beneath the foothills to ~ 58 km beneath the Higher Himalaya and then even deeper beneath southern Tibet ^31^. A mid-crustal low-velocity zone at ~ 12–18 km depth, beneath the Lesser Himalaya in western Nepal was also reported^31^. This LVZ lies in between the MBT and the MCT and is likely caused by fluids expelled from underthrust sedimentary rocks trapped at the MHT^31^. The spatial distribution of simulated crustal Vp/Vs ratios reveals a large lateral variation in the crustal composition of the Uttarakhand Himalayan region^32^. Large variations in the crust's thickness (28.3 to 52.9 km) and Poisson’s ratio (0.17 to 0.36) indicate the presence of serpentinization and high pressure metamorphic fluids in the crust^32^.

In a Lg Q_o_ attenuation study^33^ conducted along a profile in the Garhwal Himalaya area of the northwest Himalaya found Indian shield-like Q_o_ values of 742 ± 235 in the Lower Himalaya, whereas the High Himalaya is distinguished by an exceptionally low Q_o_ value of 30–60. Their results suggest the presence of a low viscosity channel in the Higher Himalaya. A similar study^34^ for the Ladakh–Karakoram and southern Tibet suggests high crustal seismic attenuation (Q_o_∼70) beneath the region with possible presence of aqueous fluid/partial melt in the Ladakh crust.

All of the above mentioned geophysical studies in the Garhwal and Kumaon region of Uttarakhand, India highlights the fact that this region has been seismically very active in the recent decades and involves a structurally complex and heterogenous crust. According to the 2011 census this region has a population density of 69.77% residing in rural villages with poor living conditions and with bare minimum services. There is also very poor enforcement of earthquake resistant building practices, which makes the region highly vulnerable to future seismic hazards of ground motion. An ability of accurately estimate ground motions from future earthquakes is needed to address the seismic hazard of a region requires estimation of ground motion from future earthquakes. This ability requires knowledge of the crustal velocity structure, characterization of earthquake source zones, and quantification of seismic wave attenuation from the source to any point on the surface of the Earth. The first two requirements have been extensively studied for the Garhwal-Kumaon Himalaya, over the past decade^26,27,35–40^ but gaps exist in our knowledge of the attenuation structure. The present study focuses on estimation of seismic wave attenuation using the Lg wave spectra from earthquakes recorded at regional distances to ascertain the quality factor (*Q*) for the crust beneath the Kumaon Himalaya, India. This should provide more constraints on assessment of future seismic hazard scenarios in the Kumaon sector of the northwest Himalayas.

## Data

CSIR-NGRI initiated a project to study the seismic hazard aspect of the Kumaon Himalaya referred to as “**S**ynthesis of Earthquake Hazard scenario in NW Himalaya by Investigating the multi-scale Variations in structural and seismotectonic Assemblages” (SHIVA). The seismic attenuation study in the Kumaon Himalaya focused on the central seismic gap zone along the SW-NE transect covering MFT, MBT, MCT and STD and used 32 broadband seismic stations that operated September-2018 to March-2022^41,42^. All the stations were equipped with CMG-3 T (120 s period) sensor and REFTEK (RT 130–01) data loggers with 4 GB swappable hard disk and GPS. Details of individual stations are presented in Table 1.

**Table 1:** List of broadband seismic stations and locations.

Station name	Latitude (°N)	Longitude (°E)	Elevation (m)
S00-Pangla	29.9784	80.6871	1557
S01-Dantu	30.2535	80.5424	3323
S02-Nagling	30.1802	80.5742	2927
S03-Sela	30.1549	80.6099	2550
S04-Bogling	30.0853	80.6221	2264
S05-Hardyala	30.066	80.5845	1841
S06-Bhethi	30.0301	80.5794	1577
S07-Gargaun	29.9774	80.5775	1333
S08-Syankuri	29.9299	80.5745	1247
S09-Dobat	29.8779	80.5624	662
S10-Nigalpani	29.8251	80.5096	831
S11-Kalika	29.8097	80.4816	1062
S12-Nagtad	29.8057	80.4443	690
S13- Kimkhola	29.7713	80.3941	777
S14-Bhagrihat	29.7454	80.3632	633
S15-Bhichatha	29.6687	80.3338	1143
S16-Mankot	29.6091	80.3387	1633
S17-Saloni	29.5316	80.2807	1326
S18-Barabe	29.5046	80.2364	1909
S19-Chathi	29.4812	80.1983	1141
S20-Mau	29.4284	80.1673	1528
S21-Kimtoli	29.39	80.1495	1794
S22-Bardoli	29.344	80.1238	1602
S23-Sayyali	29.2769	80.1221	1175
S24-Baijankhan	29.2314	80.088	1558
S25-Chalthi	29.1903	80.0945	666
S26-Sukhidang	29.1577	80.1098	1225
S27-Bastia	29.1197	80.0865	389
S28-Karkaligate	29.0767	80.0953	285
S29-Khawkote	29.7005	80.3224	1241
S30-Fagpur	29.01689	80.0925	217
S31-Chakunibora	29.3112	80.1189	1787
S32-Tidhang	30.277	80.5256	3378

**Figure 2:**
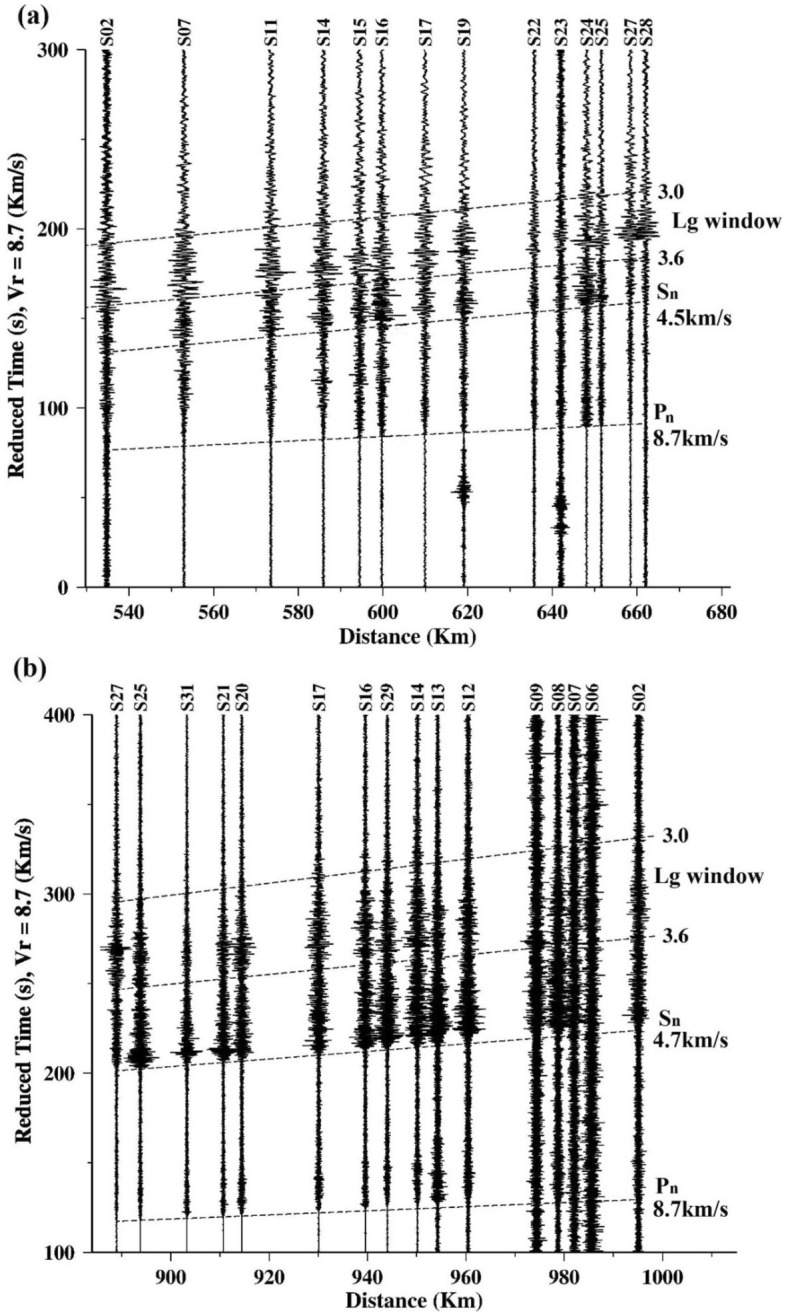
Record section of earthquakes from (**a**) Southern Tibet and (**b**) Indian Shield. Seismograms that have been high passed with corner frequency at 0.5 Hz. The Pn velocity for these two earthquakes is 8.7 km s^−1^. A Strong Lg wave is recorded in seismograms for Southern Tibet (**a**), whereas it is feeble for the Indian shield earthquake (**b**). Sn velocities for these two earthquakes are 4.5 km s^−1^ and 4.7 km s^−1^.

The Lg wave recorded on the seismograms of regional earthquakes (Table 2) are extracted with corner at 0.5 Hz using in the time window appropriate for a wave travelling with a group velocity between 3.0 and 3.6 km s^−1^^3^ (Fig. 2).

**Table 2:** Details of events used in the study.

Event ID	Date	Origin time	Latitude (°N)	Longitude (°E)	Magnitude (M_b_)
1	02/11/2018	22:35:10	34.101	83.920	4.6
2	14/11/2018	17:10:48	34.014	83.900	4.5
3	20/11/2018	21:44:54	34.064	83.940	4.4
4	05/06/2019	17:01:12	24.537	72.735	4.0
5	13/10/2019	05:06:33	27.485	76.867	4.0

**Figure 3:**
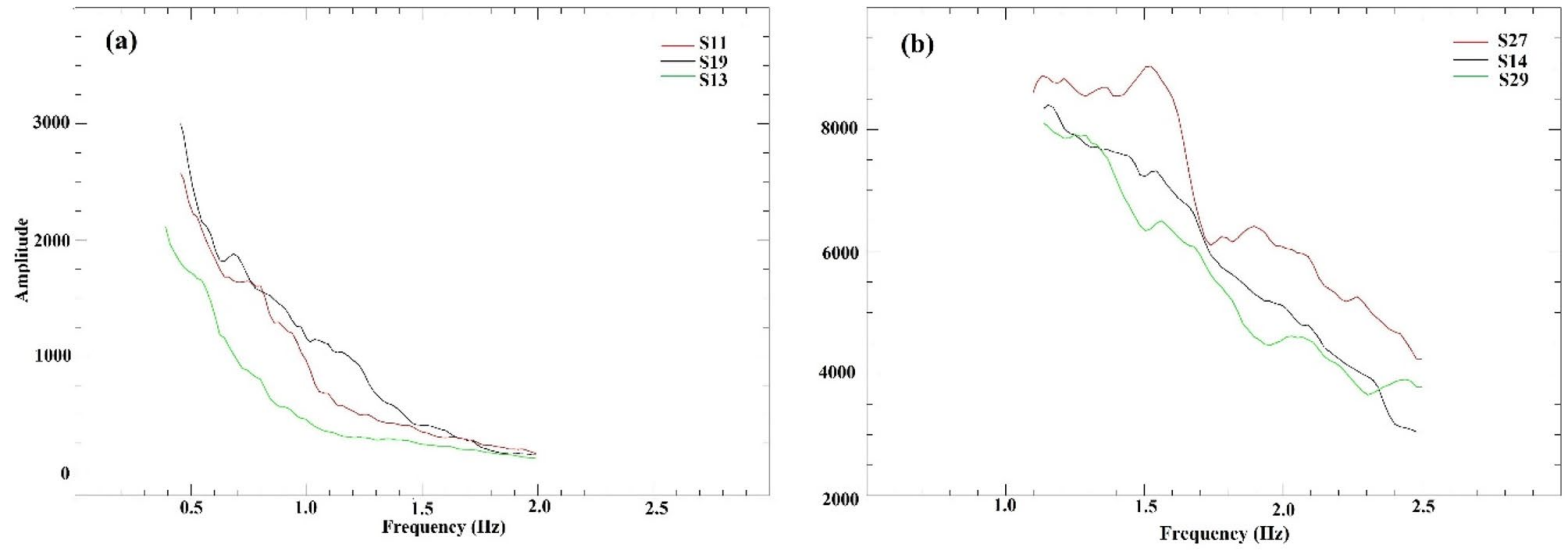
Amplitude spectra for an earthquake from (**a**) Southern Tibet on 2018 November 20 and (**b**) Indian shield on 2019 June 5 at selected stations.

The Lg wave was then cut out and a cosine taper of width 0.05 was applied to avoid spectral leakage. The instrument response was removed after transforming to the frequency domain using the fast Fourier transform (FFT). To stabilize the measurement of Q_o_, we applied a 15-point smoothing to the ground displacement spectra^3^ and computed the spectral ratio in the frequency band 0.4–2 Hz. Here we have utilized a total five regional events of which three events were in the Tibet region and two were from the Indian shield region. Figure 3a,b shows the plot of example of Lg amplitude spectra of selected stations.

## Methodology

Lg is a regional seismic wave comprised of multiple shear wave reverberations trapped in the crustal waveguide, and is important for studying the crustal structure. The Lg Q at frequencies greater than 1 Hz is observed to obey a frequency dependence power law of the form

1 $$ {\text{Q}}_{{{\text{Lg}}}} \left( {\text{f}} \right) = {\text{Q}}_{{\text{o}}} {\text{f}}^{\eta } $$

where Q_o_ and ƞ are Lg Q at 1 Hz and its power law frequency dependence, respectively. The value for Lg Q for tectonically active regions has been observed to be approximately ≤ 200 and that for stable regions the value is ≥ 650^3,20^. The quality factor (Q_o_) generally increases with frequency. We analysed the Lg Q at 1.0 Hz to maintain the uniformity within the analysis and to accurately determine the relative variation within different lithotectonic units.

### Estimation of Lg Q_o_

We have utilized the two-station technique^3^ to compute the Lg Q attenuation structure beneath the Kumaon Himalaya. If two-stations denoted as station i and station j then the method collects the Lg amplitude spectra, which is denoted as A_i_(f) and A_j_ (f) at a particular frequency, f respectively. Then we calculate the scaled amplitude ratio following the methodology^3^, which states that

2 $$ {\text{R}}\left( {\text{f}} \right) = \left( {\Delta_{{\text{i}}}^{{{1}/{2}}} /\Delta_{{\text{j}}}^{{{1}/{2}}} } \right) \, \left[ {{\text{A}}_{{\text{i}}} \left( {\text{f}} \right)/{\text{A}}_{{\text{j}}} \left( {\text{f}} \right)} \right], $$

where Δ_i_ and Δ_i_ represents the epicentral distances and the effect of geometrical spreading on the readings are cancelled by taking the square root of their ratio in 1-D computations.

Equation (2) can be written as.

3 $$  \begin{aligned}    & \left( {{\text{V}}_{{{\text{Lg}}}} /\pi \Delta _{{{\text{i}},{\text{j}}}} } \right){\text{ ln}}\left[ {{\text{R}}\left( {\text{f}} \right)} \right]\, = \,{\text{f}}^{{{\text{1}} - \eta }} /{\text{Q}}_{{\text{o}}} \quad {\text{or}} \\     & {\text{R}}\left( {\text{f}} \right) = \left( {\Delta _{{\text{i}}} ^{{{\text{1}}/{\text{2}}}} /\Delta _{{\text{j}}} ^{{{\text{1}}/{\text{2}}}} } \right){\text{ }}\left[ {{\text{A}}_{{\text{i}}} \left( {\text{f}} \right)/{\text{A}}_{{\text{j}}} \left( {\text{f}} \right)} \right], \\  \end{aligned}  $$

where P(f) = (V_Lg_/πΔ_i,j_) ln[R(f)]

V_Lg_ is the typical Lg group velocity. The Q_o_ and η values can be calculated by fitting a line using the least-squares criterion. While the computation of Q_o_ is stable, the measurement of stable interstation η is difficult. Further, due to a limited availability of earthquake-station pair measurements, we restricted this study to Q_o_ variation only. Accordingly, we used η = 0 in Eq. (3) and computed the Q_o_ along with the associated error in individual two-station data.

Initially, we selected all possible two-station pairs for the analysis to estimate the interstation spectral ratios but later on we applied the suggested criterion^3^ to examine the final two-station pairs for the Lg Q analysis.

4 $$ \delta {\text{Q}}_{{\text{o}}} /{\text{Q}}_{{\text{o}}} \approx {1}.{1}\left( {{\text{Q}}_{{\text{o}}} /\Delta_{{{\text{i}},{\text{j}}}} } \right) \, \delta {\text{x}} $$

There is another important limitation to this method, which assumes that the recording stations are aligned exactly with the source, but in a real scenario, a perfect alignment of stations and source is usually not possible^3^. To define the alignment, an angle δθ, which is the difference between the azimuths from the source and the two stations, is used. A previous study^43^ allowed δθ up to 10°, whereas another study^3^ used 15°. The amplitude spectra of two events one from the north-eastern and other from the south-western side of the profile are shown in Fig. 3a and b, respectively.

### Inversion for regional variation of Q_o_

The inversions are composed of two steps. In the primary step, interstation Q_o_ is measured from selected pairs of two stations (Table 3). In the second step, the interstation Q_o_ values are mapped using inversion. We have applied the singular value decomposition method^44^ to compute the variations of Lg Q_o_ attenuation along the profile in the Kumaon Himalaya. We have divided our NE-SW profile in to four different cells with the M denoting the discrete Lg Q_o_ values in different grids, and where N is the number of observations (Q value along a two-station path, so N = number of paths). If there are N interstation Q_o_ measurements and the region is divided into M cells, the quality factor value for individual cells (Q_m_) is related to the two-station measurements of quality factor (Q_n_) as:

5 $$ \Delta_{{\text{n}}} /{\text{Q}}_{{\text{n}}} = \, \Sigma \, \Delta_{{{\text{mn}}}} /{\text{Q}}_{{\text{m}}} + \varepsilon {\text{n}}, \,{\text{n}}\, = \,{1},{2},{3}, \ldots {\text{N,}} $$

where Δ_mn_ is the length of the nth ray in the mth cell. εn is the error for each Q_n_ measurement.

**Table 3:** Two-station Q0 measurements.

Event ID	Station pairs	Q_0_ values
1	S22-S28	33.79 ± 2
	S16-S23	42.75 ± 3
	S16-S22	66.17 ± 10
	S14-S23	81.14 ± 15
	S13-S22	83.4 ± 14
	S12-S23	58.36 ± 4
	S12-S22	86.47 ± 10
	S12-S17	47.49 ± 3
	S04-S11	25.87 ± 3
2	S05-S07	23.63 ± 5
	S07-S11	62.54 ± 31
	S02-S07	44.96 ± 6
	S02-S08	20.82 ± 1
	S03-S12	80.88 ± 7
	S04-S07	17.21 ± 1
	S04-S12	116.06 ± 26
	S07-S11	63.9 ± 22
	S13-S19	14.27 ± 1
	S13-S27	240.12 ± 79
	S13-S28	111.58 ± 22
	S14-S15	28.53 ± 6
	S14-S22	116.25 ± 34
	S16-S22	46.48 ± 7
	S17-S19	5.13 ± 1
	S17-S22	53.73 ± 18
3	S25-S27	14.32 ± 2
	S23-S28	47.09 ± 9
	S22-S28	76.82 ± 15
	S17-S19	12.92 ± 1
	S16-S22	101.66 ± 17
	S15-S19	42.39 ± 6
	S14-S22	102.43 ± 25
	S14-S17	36.83 ± 9
	S14-S16	114.19 ± 6
	S14-S15	10.92 ± 1
	S13-S27	107.31 ± 18
	S13-S22	67.94 ± 5
	S13-S19	21.32 ± 5
	S13-S14	12.14 ± 1
	S12-S22	62.8 ± 5
	S12-S17	29.88 ± 3
	S11-S22	206.97 ± 53
	S11-S17	73.3 ± 16
	S10-S22	40.18 ± 1
	S03-S08	48.85 ± 10
4	S02-S14	144.42 ± 21
	S12-S31	159.64 ± 38
	S17-S20	143.86 ± 56
	S17-S25	82.66 ± 15
	S17-S27	185.38 ± 23
	S17-S29	195.18 ± 70
	S17-S32	594.16 ± 191
	S21-S31	30.88 ± 4
	S24-S25	11.74 ± 2
	S24-S27	85.85 ± 25
5	S02-S25	465.8 ± 172
	S06-S07	14.68 ± 1
	S06-S15	165.53 ± 57
	S06-S17	105.95 ± 14
	S06-S24	372.6 ± 79
	S10-S17	97.91 ± 13
	S15-S17	69.58 ± 33

The resulting system of N equations is solved through a singular value decomposition algorithm in MATLAB to obtain the quality factor values for each individual cells. The economy-size decomposition algorithm of MATLAB removes extra rows or columns of zeros from the diagonal matrix of singular values along with the columns in either of data (Q_n_) or kernel (G) matrices that multiply those zeros in the expression

6 $$ {\text{Q}}_{{\text{m}}} = {\text{ G}}*{\text{m}}*{\text{Q}}_{{\text{n}}} $$

**Figure 4:**
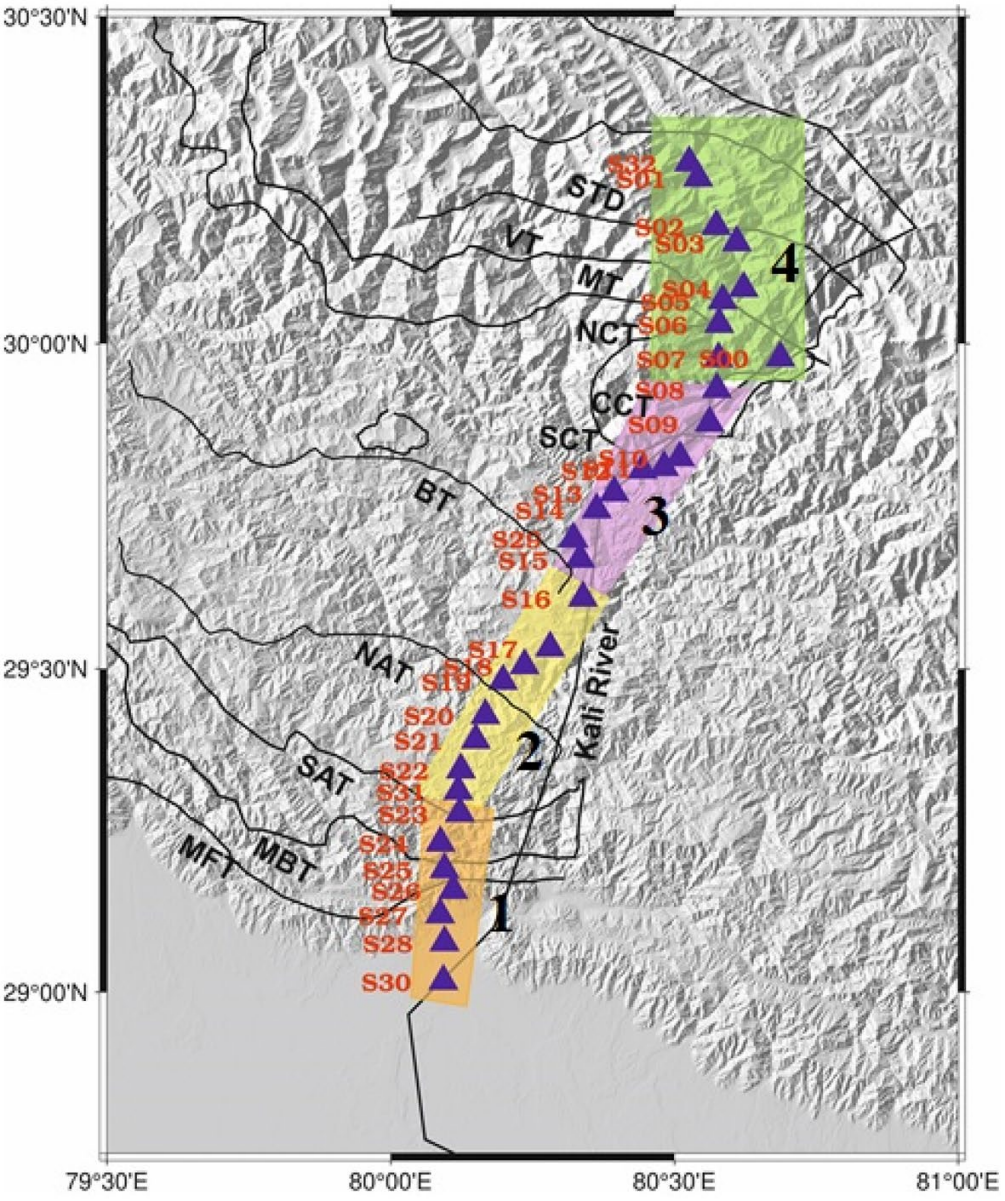
Spatial parameter used to compute the Lg Q value in different blocks N = 1,2,3,4. This figure has been generated using the GMT, version 6.0 software (https://docs.generic-mapping-tools.org/6.0/).

Removing these zeros and columns can improve execution time and reduce storage requirements without compromising the accuracy of the decomposition. The four blocks are shown in Fig. 4 with different colours.

## Results and discussion

Assessment of seismic hazard in an earthquake prone area involves quantification of its crustal velocity structure, earthquake source properties and the seismic wave attenuation in the area. The latter requires estimates of the seismic quality Q. Estimation of Q at 1 Hz (Q_0)_ of the Lg waves recorded from regional earthquakes is useful because of Q_0Lg_ is sensitive to crustal properties^3,6,19^. Lg attenuation is also an important factor in determining seismic hazard because Lg of the large amplitude at regional distances^45^. The seismic risk in the Himalaya is high as it is expected to host a Mw 8.0 or greater anytime in future^46^. The present study area, which forms the eastern part of the CSG in the Himalaya has hosted many moderate to strong earthquakes in the past with the two most significant earthquakes being the 1991 Mw 6.8 Uttarkashi earthquake and the 1999 Mw 6.6 Chamoli earthquakes. Quantifying the obvious hazard is an essential to prerequisite to reducing the seismic risk to the population in this region. We computed the Lg attenuation characteristics using five regional events with M_w_ 4.0 to 5.5 (Table 2) along the NE-SW profile of ∼200 km along the Tanakpur-Dharchula-Dharma transect in eastern Kumaon Himalaya. We utilized the interstation *Q*_0_ computation approach^3^ to quantify the attenuation at different places along the 200 km seismic profile. We find significant differences of Lg Q_o_ along the profile.

**Figure 5:**
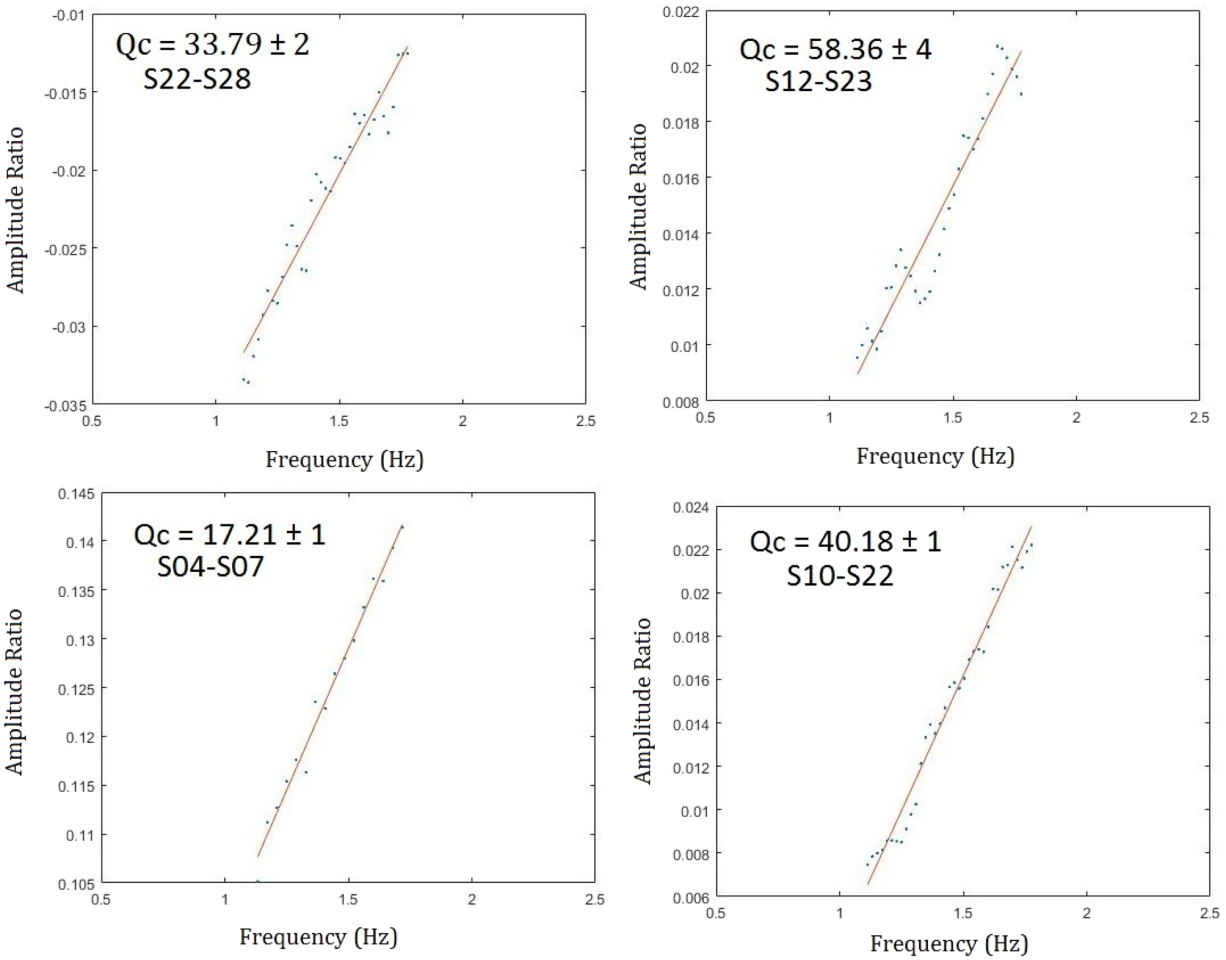
Figures showing some of the linear regression fit to compute the interstation Q_o_ values for selected source and station pair. The obtained Q_c_ and its standard deviation are also shown.

The individual Lg Q_o_ values resulting from the various station pairs are represented in Table 3 and some examples for linear regression fit for two station pairs are shown in Fig. 5.

**Figure 6:**
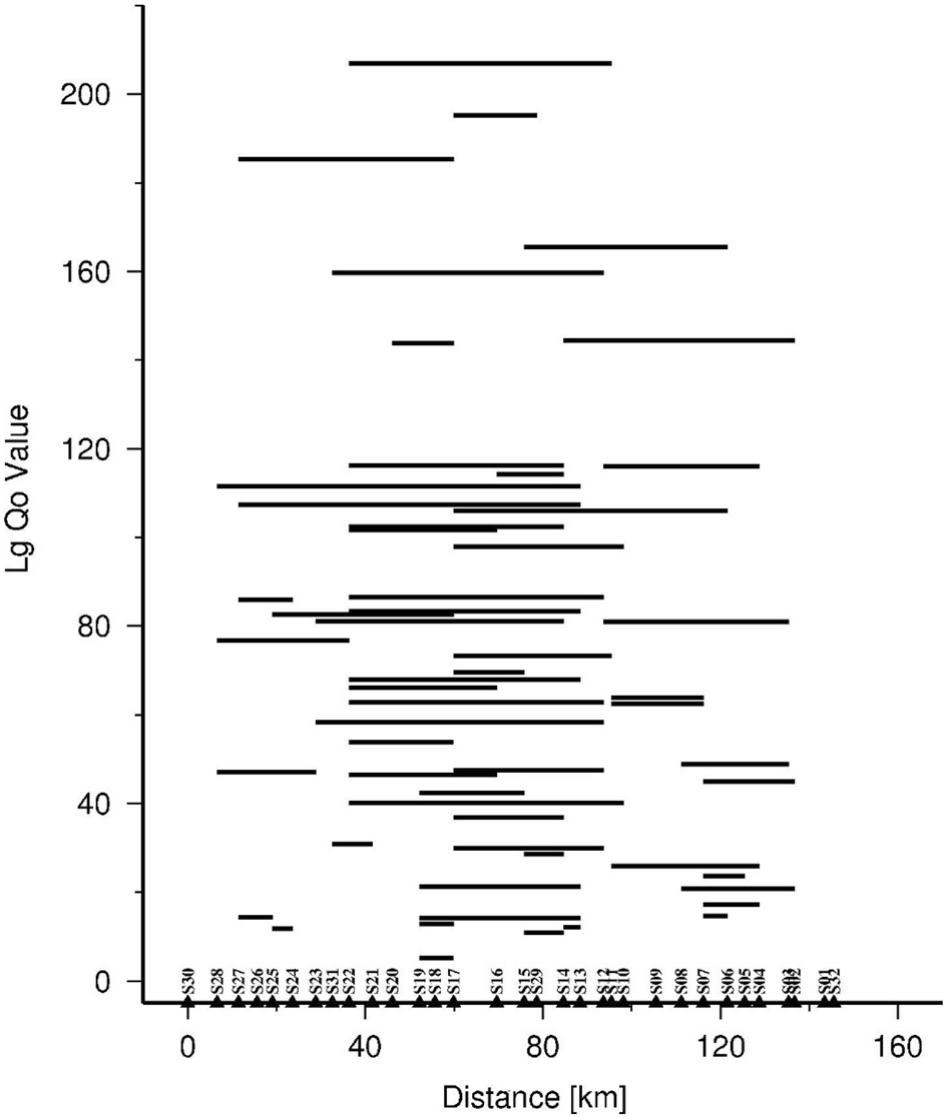
Plot of all the computed interstation Q_o_ values with different stations along the profile used for modelling the attenuation in individual parameterized cell.

The value of Lg Q_o_ from different events and different pairs of stations are plotted in Fig. 6. The Lg Q_o_ values vary 20 to 203 for 64 combinations of two stations along the profile.

**Figure 7:**
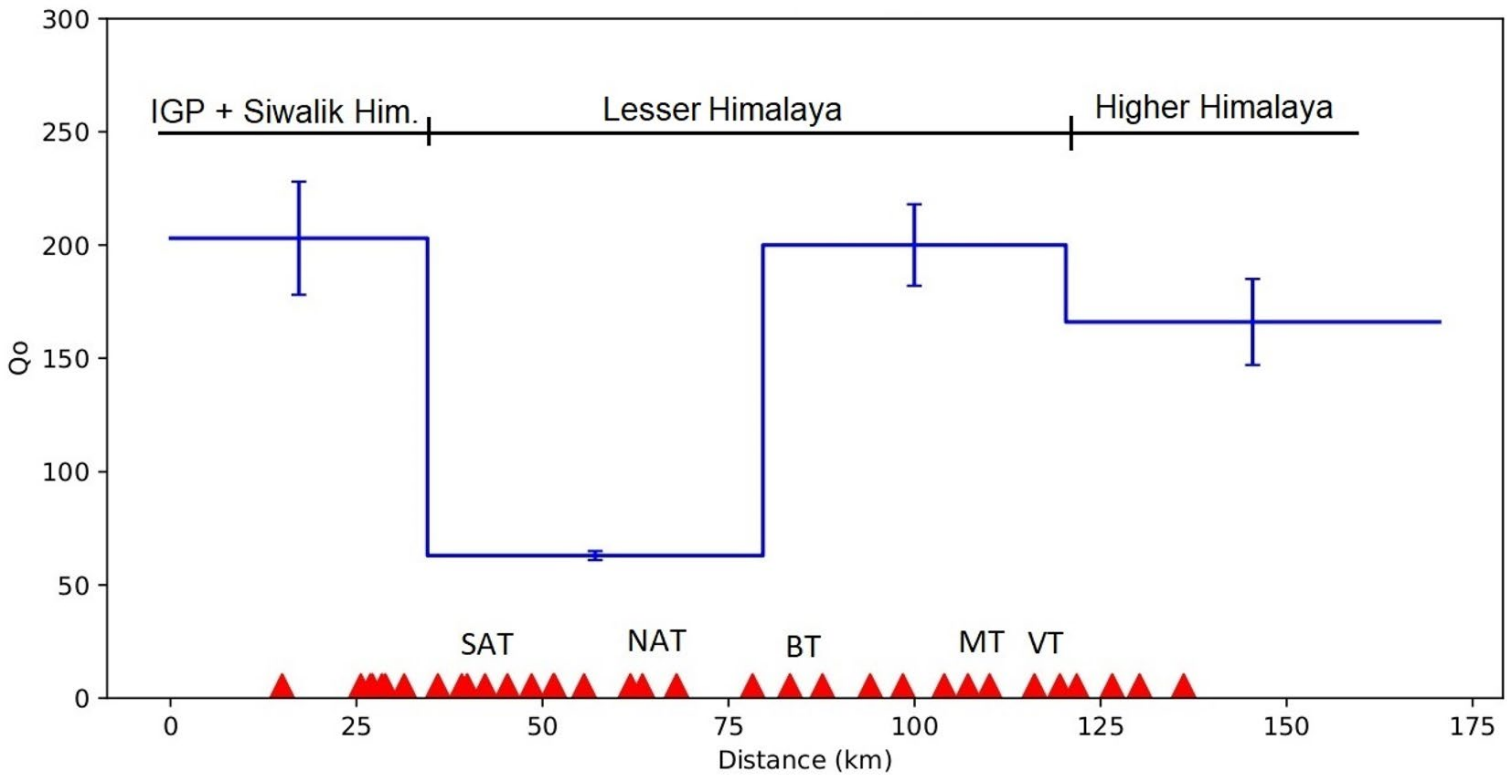
Lg Q_o_ along with its standard deviation values for the individual blocks obtained from inversion of several two-station pair data. The relative position of stations and the corresponding geological block are shown. Faults, *SAT* South-Almora Thrust, *NAT* North-Almora Thrust, *BT* Berinag Thrust, *MT* Munsiari Thrust, *VT* Vaikrita Thrust.

To better understand the lateral variability of the Q_o_ along the profile, we divide the profile into four blocks. From north to south, first block covers the IGP and Siwalik Himalaya and the second and third blocks cover the lesser Himalaya and the fourth block consists of the higher Himalaya and the Tethys Himalaya. The linear inversion of Q_o_ values computed using Eqs. (5) and (6), utilizing all the individual interstation Lg Q_o_ values (Fig. 6) resulted in a minimum value of 63 ± 2 and a maximum of 203 ± 25 for four individual blocks along the profile (Fig. 7). The Lg Q_o_ values along the profile are nearly equal to 200 for all the blocks except for a part of the second block lying between the South Almora Thrust (SAT) and the Berinag Thrust (BT), which shows characteristically low Q_o_ value of 63.

Our maximum Lg Q_o_ values resulting from the inversion of interstation Lg Q_o_ values is 203 ± 25, observed for the first block of the profile that includes some stations from Indo-Gangetic Plain, Siwalik Himalaya and some part of the Lesser Himalaya to the south of the SAT (South Almora Thrust). The reason for such low Lg Q_o_ values in the region can be attributed to the presence of younger tertiary sediments, which has characteristically low Vp/Vs ratios and cause decay of Lg wave amplitude below the paired stations. Another possibility is the lateral variation of crustal structure and sedimentary thickness (2–5 km)^47^.

This second block in the profile shows the low value of Lg Q_o_ 63 which reflects strong attenuations which consists of major secondary fault structures of the lesser Himalayan tectonic units and is mainly bounded between the South Almora Thrust (SAT) in the south and the Berinag Thrust (BT) in north. The low value of Q_o_ correlates with low resistivity and a low seismic velocity zone^28,29,48^ detected to the west.

We observed higher Q_o_ values of 200 towards the north of the BT in the third block of the profile bounded by BT in the south and Chippalkot thrust (CT) of the Dharchula area in the north. To the north of the CT, we observed very little decrease in the Q_o_ value which is equal to 166 for the fourth block. This block extends between the CT and the South Tibetan Detachment fault (STD) and consists of two seismic stations towards the north of the STD in the Tethys Himalaya. The Q_o_ values observed along this Tanakpur-Dharchula-Dharma NE-SW profile are similar to values observed along the INDEPTH III profile in Tibet by many researchers in the past^3,49–51^. The Lg Q_0_ values observed along the present profile depicts values between 63 and 203 respectively for the four different blocks in the Kumaon sector of the northwest Himalayas. The INDEPTH III experiment, which was conducted in the central Tibet region, extends further to the northeast of the present profile across the Bangong-Nujiang Suture (BNS). This area has been studied previously for seismic attenuation using the Fourier spectra of the Lg waves^3^. The results show low Lg Q_0_ values with higher attenuation characteristics, with an average Q_0_ value of ~ 158 for the entire profile length of ~ 200 km. The resultant high attenuation along the profile can be compared to the Lg Q_0_ values obtained for the INDEPTH II profile for the Southern Tibet region traversing the Indus-Yalong Suture to the north and south and extending further south into the Himalayas. Though the study in Southern Tibet and further south in the High Himalayas shows higher Lg Q_0_ values of ~ 300, these values are associated with large errors due to sparse path coverage as reported in the study^3^. The low Lg Q_0_ values of ~ 191 obtained to the north of the Indus-Yalong Suture in Southern Tibet are comparable to what we have obtained for our Higher Himalaya region in the Dharma sector of the Kumaon Himalaya. The study reports a gradual increase in the Lg Q_0_ values to the south of the Indus-Yalong Suture, which can be further explained in terms of underthrusting of the Indian Lithosphere beneath the Tibet^6^. Very low Q_o_ values are also reported for the Higher Himalaya to the north of the MCT zone^33^, which lies further to the northwest of the present profile in the Garhwal Himalaya region. The observations of such high attenuation in the Higher Himalaya can be attributed to the presence of a low velocity zone in the High Himalaya. The present study also supplements the study conducted earlier^33^ for the Higher Himalayas section of the Garhwal Himalaya and further confirms the presence of the low viscous partial melts in the form of Miocene leucogranite plutons, which resulted out of the Indo-Asian collision. Another significant study^34^ along a profile from the southern edge of the northwestern Himalaya to Ladakh and Karakoram indicates some interesting lower Lg Q_0_ values, which gradually decreases northwards from ∼700 in Himalaya and ∼400 beneath the Indus Tsangpo suture (ITS) to ∼70 in Ladakh–Karakoram. The study further suggested an efficient transmission of seismic waves beneath the Himalayas and the IZS as compared to the high attenuation under Ladakh–Karakoram. It is impossible to postulate high Lg Q_0_ beneath High Himalayas as the crust beneath that is extremely fractured due to ongoing convergence between the India and the Eurasia plates. A significant study to the east of the Ladakh Himalaya using coda waves reported an average Qc between 47 for a 10 s lapse time with a 10-s coda window and 204 for a 50 s lapse time and 50 s window length^52^. Those coda Q values are similar to the Lg Q_0_ values found in the present study, which range between 63 and 200. The study conducted for the NW Himalaya^52^ as well as the present study find results similar those conducted in the adjoining Western Himalaya^53,54^, which report a low Q_0_ ≈ 44 and Q_0_ ≈ 74 respectively.

The study^38^ investigated the seismotectonic perspective for the Kumaon Himalaya and reported a complex faulting pattern, which is correlated with our reported low Q0 values to the south of the VT in the MCT zone. The reason for observing such structural or crustal scale heterogeneities can be ascribed to the presence of fluid-rich zone as well as strain localization and large stress build-up due to the locking in the ramp structure on the MHT in the Dharchula or the Chipalkot crystalline belt. We believe that the ramp structure extends further south into the region between BT and SAT in the Inner lesser Himalayas and leads to lowering of the Q_0_ values in this part of the lesser Himalayas.

Another significant study^39^ utilizing the P-wave receiver functions reported the presence of a ramp in the crust, which dips at about ∼20°. This ramp or duplex structure is responsible for introducing crustal heterogeneities within the crust. The study also reports the presence of a low velocity layer in the lesser Himalaya with aqueous fluids that can result in high attenuation of the Lg spectra below the lesser Himalayan crust. The presence of aqueous fluid is well correlated with the mechanism of decompression melting^55^, which can be associated with the melting of the lower crust in the region.

High Q_o_ were reported for the southern part of the Lesser Himalaya zone between MBT and MCT in the Garhwal section, to the northwest of the present profile^33^.

For our profile, we observed a characteristically low Q_o_ values and high attenuation in the range from 63 to 200 for the Lesser Himalayan tectonic unit, which lies between the two crustal scale faults MBT and the MCT. Our observations in the Higher Himalaya tectonic unit are similar to those from the previous study^33^, which has also reported low Lg Q_0_ values between 30 and 60 in the actively deforming Higher Himalaya. To the south of the MCT zone in the Lesser Himalaya they have reported high Lg Q_0_ values of 742 ± 235, which also shows a large error associated with the value and this is not an acceptable value for the actively deforming Himalayas. This large error reported for the Lesser Himalaya section in Garhwal Himalaya^33^ does not support the underthrusting mechanism of the Indian plate beneath Himalayas, which makes the region seismically very active and contradicts the presence of a stable lithosphere like the Indian Shield beneath the Himalayas.

Our findings show low Q_o_ values and high attenuation for the Lesser Himalayan tectonic unit in accordance with LVL and low resistivity in between MBT and MCT^29^. A previous study of this region also reported high Poisson’s ratio due to the presence of fluids/partial melts at mid-crustal depths, which in turn controls the rheological strength of the crust leading to high crustal attenuation in the region^32^. The presence of fluids beneath the Higher Himalaya and the Tethys Himalaya tectonic unit has led to the long-term structural and compositional evolution of the active fault zones through decrease in fault friction^28^. This decrease in frictional strength of the fault causes a more heterogeneous fault behaviour and leads to high seismic attenuation of the L_g_ waves^56–58^. The Tethys Himalaya north of the STD is reported to be actively deformed and consists of abundant normal faults with east–west extension^59^, which can lead to the presence of strong small-scale structural heterogeneities within the crust that might have also contributed observed low Lg Q_o_ values in the region.

**Figure 8:**
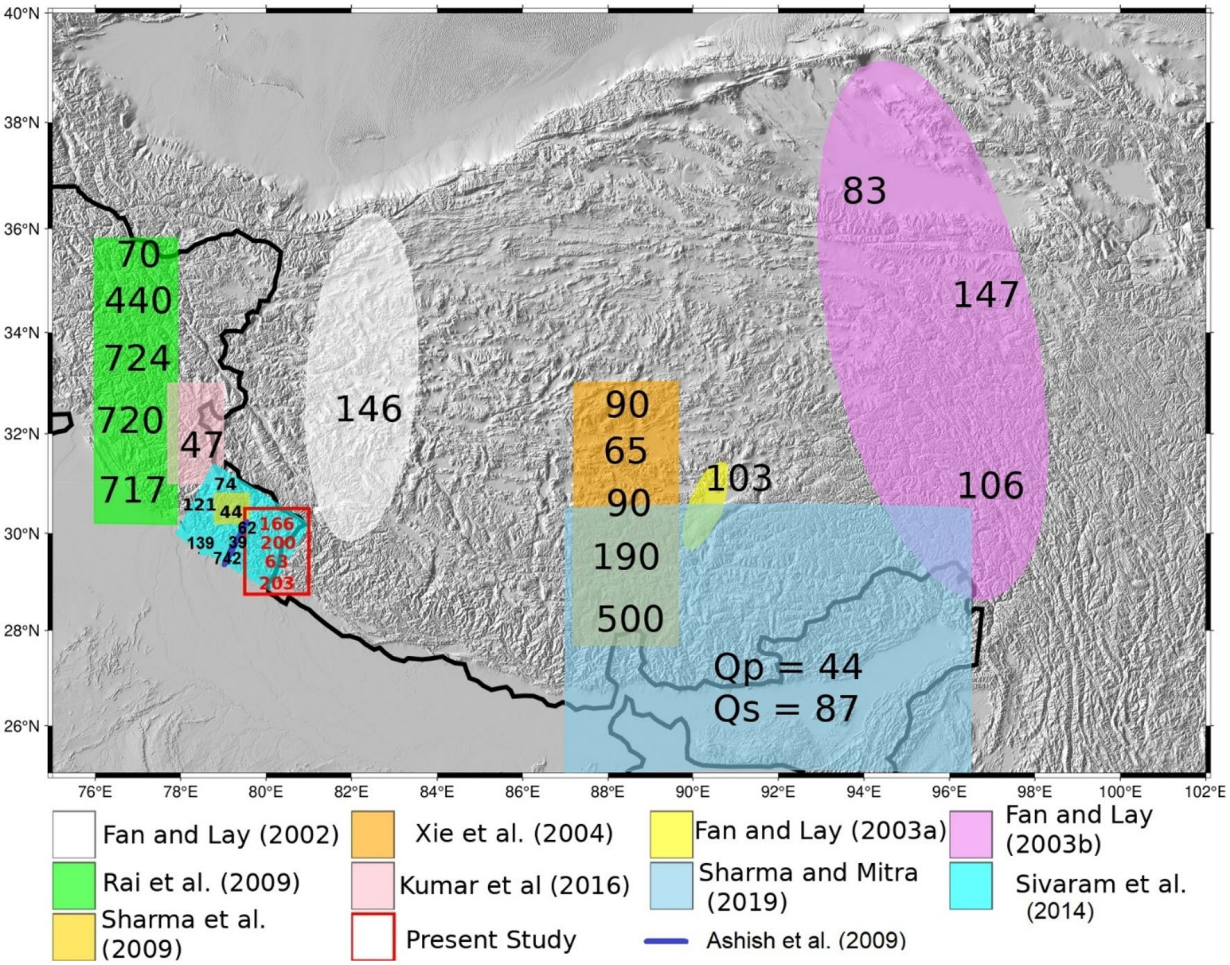
Map showing the comparison plot for the attenuation studies carried out in different part of the Himalaya-Tibet (References there-in). Figure generated using the GMT, version 6.0.

There have been other studies available from the investigation of seismic attenuation utilizing different types of waves for the Himalaya-Tibet region (Fig. 8). Similar results of lower Q_p_ (≈ 44) and Q_s_ (≈ 87) at 1 Hz utilizing body waves were reported for the northeast Himalaya^60^. The results from most of the seismic attenuation studies reports low Q_o_ values for the Himalaya-Tibet orogenic belt and the possible reasons include presence of crustal heterogeneities and trapped partial melts in the mid-crust^61^. Another significant reason controlling the seismic Q factor beneath Himalaya can be due to the crustal thickening and presence of thick sedimentary layers of 2–5 km^47^ below the Himalaya and the adjoining Indo-Gangetic Plain. The results obtained here for the Himalaya are similar to those found for other active seismic zones of the world.

## Conclusions

Lg spectra recorded along the Tanakpur-Dharchula-Dhrama transect is utilized to study the seismic attenuation or the Q structure at 1 Hz beneath the western Kumaon sector of the NW Himalaya. A two-station method and a back projection algorithm are used to obtain models of laterally varying *Lg Q*_0_ (*Lg Q* at 1 Hz). This resulted in Q_0_ values to be low (∼ 63 -203) for the entire profile. We interpret these extremely low Lg Q_0_ values in the Kumaon Himalaya region being caused by the presence of crustal heterogeneities due to ramp structures and/or trapped fluids/partial melts caused due to high temperature in the crust.

Our results are consistent with the previous findings of strong attenuation beneath Higher Himalaya and Tethys Himalaya in Tibet as well as other sections of the northwest and central Himalayas but somehow differs for the Lesser Himalaya, which earlier reported low attenuation. Though this study has been conducted with a good quality of seismic datasets but there is a scope of obtaining Lg Q_0_ tomographic images along the profile, which would be more useful in having better constraints on the crustal velocity structure variations in the actively deforming Himalaya collision zone.

## Data Availability

The raw seismic waveforms used and/or analyzed during the current study is available from the corresponding author on reasonable request through the CSIR-NGRI competing authority. The figures presented in the manuscript has been generated using the Generic Mapping Tool (GMT), version 6.0 (https://docs.generic-mapping-tools.org/6.0/). The regional variation of the Q_0_ structure has been obtained utilizing the MATLAB, version R2018a (academic license for CSIR-NGRI).
