# Author Correction: Delineation of partial melts and crustal heterogeneities within the crust beneath Kumaon Himalaya, India from L_g_ wave attenuation

**DOI:** 10.1038/s41598-023-39351-8

**Published:** 2023-07-27

**Authors:** Mahesh Prasad Parija, Sudesh Kumar, Arjun V. H

**Affiliations:** 1 CSIR-National Geophysical Research Institute, Hyderabad, India; 2 Department of Geosciences, Virginia Tech, Blacksburg, VA USA; 3 National Center for Polar and ocean Research, Ministry of Earth Sciences, Headland Sada, Vascodagama, Goa 403804 India

Correction to: *Scientific Reports* https://doi.org/10.1038/s41598-023-36269-z, published online 19 June 2023

In the original version of this Article, Sudesh Kumar was omitted as a corresponding author. Correspondence and requests for materials should also be addressed to kumarsudesh77@gmail.com

The original Article has been corrected.

